# Author Correction: Metagenomic sequencing detects human respiratory and enteric viruses in air samples collected from congregate settings

**DOI:** 10.1038/s41598-024-51814-0

**Published:** 2024-01-15

**Authors:** Nicholas R. Minor, Mitchell D. Ramuta, Miranda R. Stauss, Olivia E. Harwood, Savannah F. Brakefield, Alexandra Alberts, William C. Vuyk, Max J. Bobholz, Jenna R. Rosinski, Sydney Wolf, Madelyn Lund, Madison Mussa, Lucas J. Beversdorf, Matthew T. Aliota, Shelby L. O’Connor, David H. O’Connor

**Affiliations:** 1 Wisconsin National Primate Research Center, Madison, WI USA; 2 Department of Pathology and Laboratory Medicine, University of Wisconsin-Madison, 555 Science Drive, Madison, WI 53711 USA; 3 City of Milwaukee Health Department Laboratory, Milwaukee, WI USA; 4 Department of Veterinary and Biomedical Sciences, University of Minnesota, Twin Cities, Minneapolis, MN USA

Correction to: *Scientific Reports* 10.1038/s41598-023-48352-6, published online 04 December 2023

The original version of this Article contained an error in the Abstract.

"Throughout the COVID-19 pandemic, environmental surveillance strategies, including wastewater andair sampling,"

now reads

"Throughout the COVID-19 pandemic, environmental surveillance strategies, including wastewater and air sampling,"

The original Article has been corrected.

